# Pressure-Induced Fibroid Ischemia: First-In-Human Experience with a Novel Device for Laparoscopic Treatment of Symptomatic Uterine Fibroids

**DOI:** 10.1007/s43032-022-01033-7

**Published:** 2022-08-08

**Authors:** Michael G. Tal, Ran Keidar, Gilad Magnazi, Ohad Henn, Jin Hee Kim, Scott G. Chudnoff, Kevin J. Stepp

**Affiliations:** 1 Division of Interventional Radiology, Hadassah Medical Center, Jerusalem, Israel; 2 Department of Obstetrics and Gynecology, E. Wolfson Medical Center, Holon, Israel; 3 Sackler Faculty of Medicine, Tel-Aviv University, Tel-Aviv, Israel; 4 Empress Medical Ltd., Tel Aviv, Israel; 5 Department of Obstetrics & Gynecology, Columbia University, New York, NY USA; 6 Obstetrics and Gynecology, Maimonides Medical Center, New York, NY USA; 7 Atrium Health Women’s Care Urogynecology and Pelvic Surgery, Atrium Health, Charlotte, NC USA

**Keywords:** Fibroid, Ischemia, Pressure, Suturing

## Abstract

The purpose of this study was to assess the feasibility of use of a novel uterine fibroid treatment device hypothesized to cause fibroid infarction by increasing intra-tumoral pressure. Between August 2019 and January 2020, 21 uterine fibroids were treated in 16 symptomatic pre-menopausal black women. Pelvic magnetic resonance imaging was performed before the procedure, a day after the procedure and at 1, 3, 6, and 12 months. The subjects were also followed for clinical outcomes and quality of life up to 12 months at a single investigational site. At 3 months, the mean reduction in the fibroid volume was 36.3% (*P* = .002). Incremental reduction in volume peaked at the end of the follow-up, at the 12-month mark (60.4%; *P* = .008). There were no procedures in which the users failed to perform laparoscopic pressure suturing of fibroids with the pressure-induced fibroid ischemia device. Improvement in the quality of life was evident in the Health-Related Quality of Life total, Energy/Mood, Control, and Sexual Function domains of the Uterine Fibroid Symptom and Quality of Life questionnaire at 3 months post-procedure. Unanticipated risks were not identified. Serious adverse events were not identified. The initial clinical assessment of the pressure-induced fibroid ischemia device supports feasibility of the approach and does not reveal serious safety concerns. Trial is currently being registered retrospectively (This was a feasibility study and therefore registration was not mandatory).

**Supplementary Information:**

The online version contains supplementary material available at 10.1007/s43032-022-01033-7.

## Introduction

Uterine fibroids (UFs), or leiomyomata, are common benign smooth muscle tumors of uncertain etiology with known associations with race/ethnicity, parity, and a suspected link to age at menarche [1, 2]. UFs are estimated to occur in over 70% of women by the onset of menopause [3–5], to be clinically apparent in 25% of women of reproductive age, and to prompt treatment in about 25% of symptomatic patients due to severity of symptoms [6–8]. The latter may include profound bleeding and anemia, pelvic pressure or pain, urinary frequency, abnormal bowel function, pain with intercourse, and effects on fertility and pregnancy outcomes [9]. In the United States (US), over 588 000 women aged 25–54, 0.92% women in this group according to the 2010 Census, seek treatment for a new diagnosis of symptomatic fibroids each year [10]. The true frequency of UFs is likely underestimated, as asymptomatic or gradually symptomatic cases may remain undiagnosed [11–13]. Incidence and prevalence numbers depend heavily on the differences in the method of diagnosis and the population assessed [13–21], but the incidence in black women living in the US is substantially higher than in the Hispanic, Asian, and white women, regardless of the diagnostic method [22]. Earlier age at the time of onset with larger and more numerous tumors in black women [5, 9, 23–26] contribute to their higher sensitivity of self-report than white women [8] and to their increased representation in the group of US women who experience associated symptoms or health concerns [27, 28]. Finally, while black and white women under 35 years of age have comparable fibroid growth rates, as measured by analysis of fibroid volume in a series of magnetic resonance imaging (MRI) scans over 12 months, these rates decline with age for white but not for black women [29]. A similar difference between the two races is also observed in the odds of a tumor growing more than 20% in 6 months [29].

**Fig. 1:**
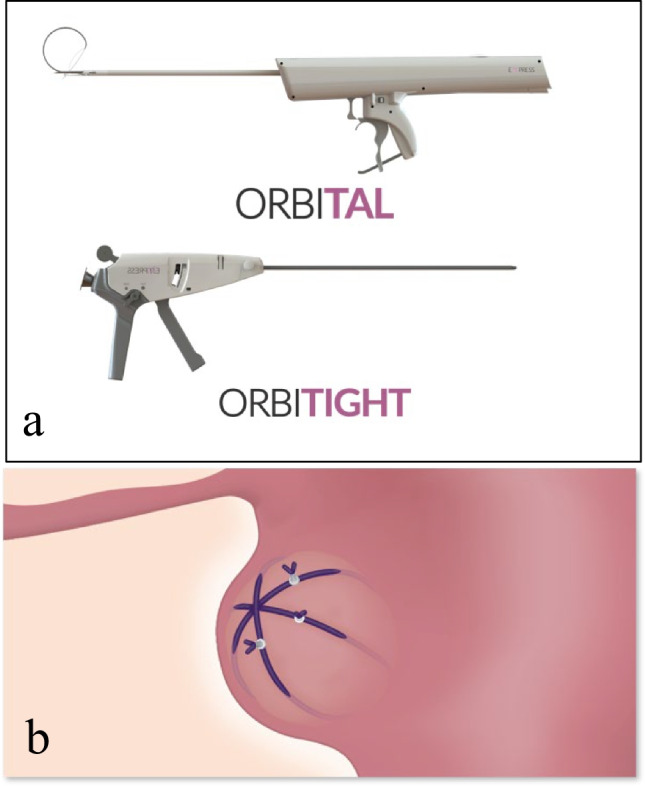
Illustration of **a** the PIFI system and **b** the mechanism of the treatment action

The pressure-induced fibroid ischemia (PIFI) system (Empress Medical Ltd., Tel Aviv, Israel; Fig. 1) is intended to treat symptomatic uterine fibroids, in a laparoscopic procedure, by increasing the intra-tumoral pressure which leads to blockage of intra- and peri-fibroid blood flow and, eventually, necrosis of the fibroid tissue. The concept of temporary blood flow occlusion with subsequent necrosis of UFs is not new and has already been successfully reduced to practice with the uterine artery embolization (UAE) approach [30] in the last quarter century. The core of this concept is the ability of the uterus to endure multiple ischemia–reperfusion cycles [31] and the fact that clots formed during flow occlusion in the fibroid blood vessels do not undergo lysis, turning transient ischemia to tumor cell necrosis [32, 33]. This study assessed feasibility of use of the PIFI system in treatment of symptomatic uterine fibroids through clinical follow-up and sequential MRI evaluation of UF size in a series of black women.

## Materials and Methods

This was a prospective, non-randomized, open-label study conducted in line with the principles of the Declaration of Helsinki in a single center in the Dominican Republic. Following the institutional ethics committee approval (Comite de Etica de Investigacion, Clinical Canela), 16 women were recruited between August 01, 2019 and January 15, 2020. Informed consent was obtained from all participants. Surgeries were performed by five laparoscopic gynecologists. Magnetic resonance imaging (MRI) results were assessed by a radiologist. The last subject completed her last visit on May 05, 2021.

### Subjects

Pre-menopausal women 30–50 years of age diagnosed with 2- to 6-cm UFs classified as type 2, 3, 4, 5, 6, or 2–5 according to the International Federation of Gynecology and Obstetrics (FIGO) subclassification system [34] and indicated for hysterectomy were recruited to the study. All subjects had to be able to understand and willing to sign the consent form. Main exclusion criteria were prior myomectomy or any surgical or minimally invasive treatment for UFs, including UAE, radiofrequency ablation, cryoablation, or MRgFUS. Other exclusion criteria were known or suspected pregnancy or future pregnancy intent, history of lower abdomen surgery, ongoing anticoagulant therapy, any life-threatening condition, and contraindication for MRI with gadolinium contrast.

### Laparoscopic Setting

Subjects lain in dorsal lithotomy position; arms secured at the sides, shoulder pads in Trendelenburg position during laparoscopy. Surgeon on the subject’s left, assistant on the subject’s right; facing subject’s legs. Laparoscopic tower at the midline of subject’s legs.

Uterine manipulators and Foley catheters were routinely inserted after patient preparation. Pneumoperitoneum was achieved using a Veress needle insufflation. A 10-mm laparoscope was introduced through an 11-mm umbilical port. Ancillary instruments were introduced through 5- or 11-mm ports. Ports were placed at the left and right lower quadrants and at a midline supraumbilical location. Pressure was maintained at 15 mmHg CO_2_ during insertion and at 12 mmHg during laparoscopy. Harmonic scalpel and/or bipolar/monopolar instruments were used if dissections were needed.

### The PIFI Procedure

To achieve its goal, the PIFI system comprises 2 handheld devices, a titanium ring, and an off-the-shelf size-0 braided, absorbable polyglactin 910 suture.

The battery-powered OrbiTal comprises a handle with a set of controllers, a tenaculum grasper located at the distal end of the 30-cm long device shaft, and a mechanism positioned in vicinity of the grasper which allows OrbiTal-firmware–controlled staged exposure of a telescopic assembly of three differently shaped needles. OrbiTal is inserted into the abdominal cavity laparoscopically, via a 5- to 8-mm trocar, and allows the surgeon to pass the suture around a fibroid. To achieve this task, once tissue is stabilized with the OrbiTal grasper, (1) the 12–G 12-mm long main (straight) needle of OrbiTal punctures the tissue; (2) the 15-G curved needle (available in 3 sizes – 32, 45, and 60 mm in diameter) circumnavigates the target fibroid under the guidance of an embedded laser pointer; (3) the 23-G 60-mm long snare-shaped needle completes encirclement of the tumor. Once the snare-shaped needle exits the tissue, the embedded suture is threaded through the snare with a laparoscopic grasper and the needles are retracted by the operator in reverse order pulling one end of the suture back to the main needle entry point.

The manually operated OrbiTight carrying the titanium ring is inserted thereafter, also through a 5- to 8-mm trocar. The suture that was wrapped around the fibroid with OrbiTal is threaded through the ring, and tightened to a pre-set tension pressure using a ratchet mechanism within OrbiTight. The ring is then crimped by OrbiTight and left on the outer surface of the uterus. Several sutures can be placed to provide adequate compression on each fibroid (Fig. 1b). Adequacy of compression was confirmed by observing the tissue affected by the suture(s), including tissue color change (whitening).

### Radiologic Follow-Up

Uterine fibroids were identified on ultrasound scans and uterine and fibroid characteristics were assessed from gadolinium-enhanced MRI (MagSense 360; Mindray Bio-Medical Electronics Co., Ltd., Shenzhen, China) 1 day and 1, 3, 6, and 12 months after the procedure. Coronal, sagittal, and transverse planes were captured. Fibroid volumes were calculated from fat-suppressed T1-weighted images by applying the prolate ellipsoid formula (π/6 multiplied by maximal longitudinal, anteroposterior, and transverse diameters).

### Primary and Secondary Outcomes

The primary study efficacy endpoint was the percentage of reduction in the treated fibroid volume 3 months after the study procedure. The primary safety endpoint was the incidence of serious adverse events (SAEs) occurring during the procedure and the 1-year follow-up. Secondary endpoint was the technical success rate, defined as the ability to apply pressure on the treated fibroid.

### Additional Outcomes

Uterine Fibroid Symptom and Quality of Life (UFS-QOL) questionnaire was collected 1, 3, 6, and 12 months after the procedure.

### Safety Analysis

All adverse events that occurred until the end of the follow-up, whether considered related to the study device and/or procedure or not, were reported.

### Statistical Analysis

Continuous variables were summarized by a mean, standard deviation, minimum, and maximum, and categorical variables by a count and percentage. Two-tailed repeated measures *t*-test was used for comparison of means (continuous variables).

Last observation carried forward (LOCF) method was used for the UFS-QOL questionnaire with available post-baseline data. Subject was excluded from the analysis for the outcome if post-baseline values were missing.

## Results

**Fig. 2:**
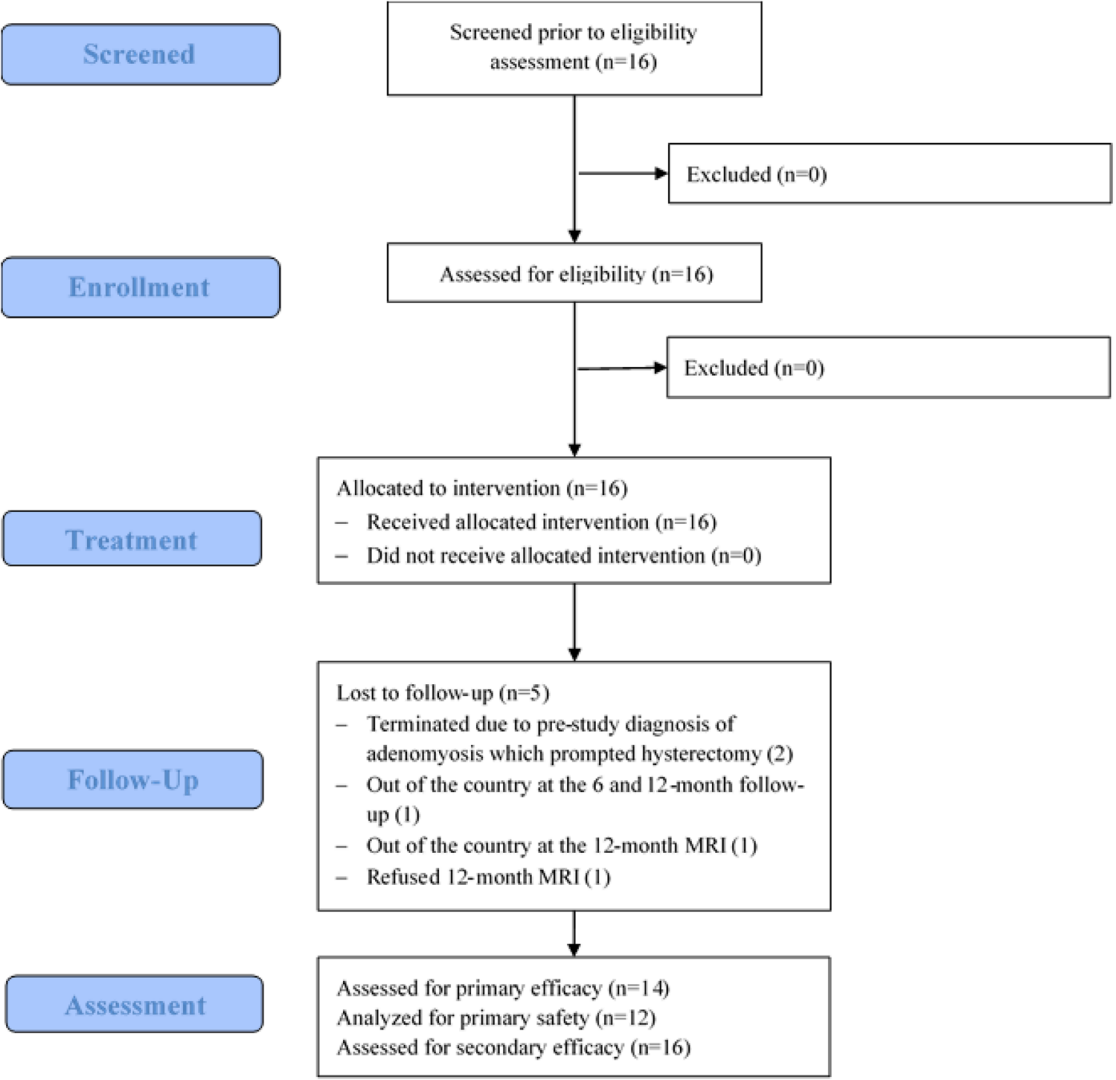
Consolidated Standards of Reporting Trials (CONSORT) flow diagram

Sixteen subjects signed informed consent for the study (Fig. 2). All women in the study were black, although this was not an eligibility requirement. Demographic and baseline characteristics of the subjects are summarized in Table 1. The mean age of subjects was 39.1 years (range, 33–49; *SD* = 4.5) and the mean body mass index (BMI) was 31.93 (*SD*, 5.18; range, 24.6–44.3). The mean per-patient number of UFs was 2.29 (range, 1–4; *SD* = 1.07). All sixteen were enrolled in the study but MRI-evaluable tumors were not identified during data analysis in one subject. In the other 15 subjects, 1–3 eligible fibroids (21 tumors overall) were treated with the PIFI system (Table 1). Participation of two subjects in the study was terminated early, at 3 and 12 months after the study procedure, due to pre-study diagnosis of adenomyosis which prompted hysterectomy. Two additional subjects were lost to follow-up and one subject refused to undergo the 12-month MRI and was withdrawn from the study. Only 5 subjects were able to arrive for their scheduled 6-month follow-up due to the travel restrictions imposed during the COVID-19 pandemic. Consequently, the 6-month timepoint is omitted from this report.

**Table 1:** Demographic and baseline characteristics of subjects

Age	Weight	Height	BMI	Gravida	Para		Abortus		Number of treated fibroids	Location of treated fibroids	FIGO classification
					Live birth	Stillbirth	Therapeutic	Miscarriage			
38	89	167	31.9	2	SVD – 1	–	1 (ectopic)	–	1	Anterior	5
33	85	162	32.4	1	SVD – 1	–	–	–	2	Anterior, left, cranial	5
										Posterior, left	6
39	95	162	36.2	3	C-section – 2	–		1	2	Anterior	4
										Right	4
36	72	160	28.1	5	SVD – 4	1	–	–	1	Right, anterior	4
49	67	156	27.5	3	SVD – 2 C-section – 1	–	–	–	–**	–**	**
35	87	160	34.0	–	–	–	–	–	1	Posterior, right	2–5
39	80	160	31.3	4	SVD – 3	–	–	1	2	Anterior, right	2–5
										anterior, left	2–5
40	59	147	27.3	1	SVD—1	–	–	–	1	Anterior, cranial	4
36	82	160	32.0	–	–	–	–	–	1	Anterior, left, cranial	4
42	71	160	27.7	–	–	–	–	–	2	Anterior, cranial	2–5
										Anterior, caudal	5
40	96	167	34.4	–	–	–	–	–	3	Left, anterior	4
										Posterior, cranial	6
										Right, caudal	6
48	122	166	44.3	1	C-section – 1	–	–	–	1	Anterior, caudal	3
40	80	155	33.3	1	SVD – 1	–	–	–	2	Right, anterior	4
										Posterior	4
35	56	151	24.6	2	C-section – 2	–	–	–	1	Anterior	5
34	75	169	26.3	2	C-section – 2	–	–	–	1	Anterior	5
41	108	165	39.7	2	C-section – 2	–	–	–	1	Anterior	2

*BMI*, body mass index; *SVD*, spontaneous vaginal delivery

**A fibroid was observed on ultrasound and visualized during the procedure, but its volume could not be reliably estimated using MRI scans

On average, 3.5 sutures (*SD* = 1.6; range, 2–6) were placed in each subject. Mean time in surgery, from trocar placement to trocar site closure, was 80 min (*SD* = 36; range, 30 – 120). There were no procedures in which the surgeons failed to perform laparoscopic pressure suturing of fibroids with the PIFI system, placing the technical success rate at 100%.

**Fig. 3:**
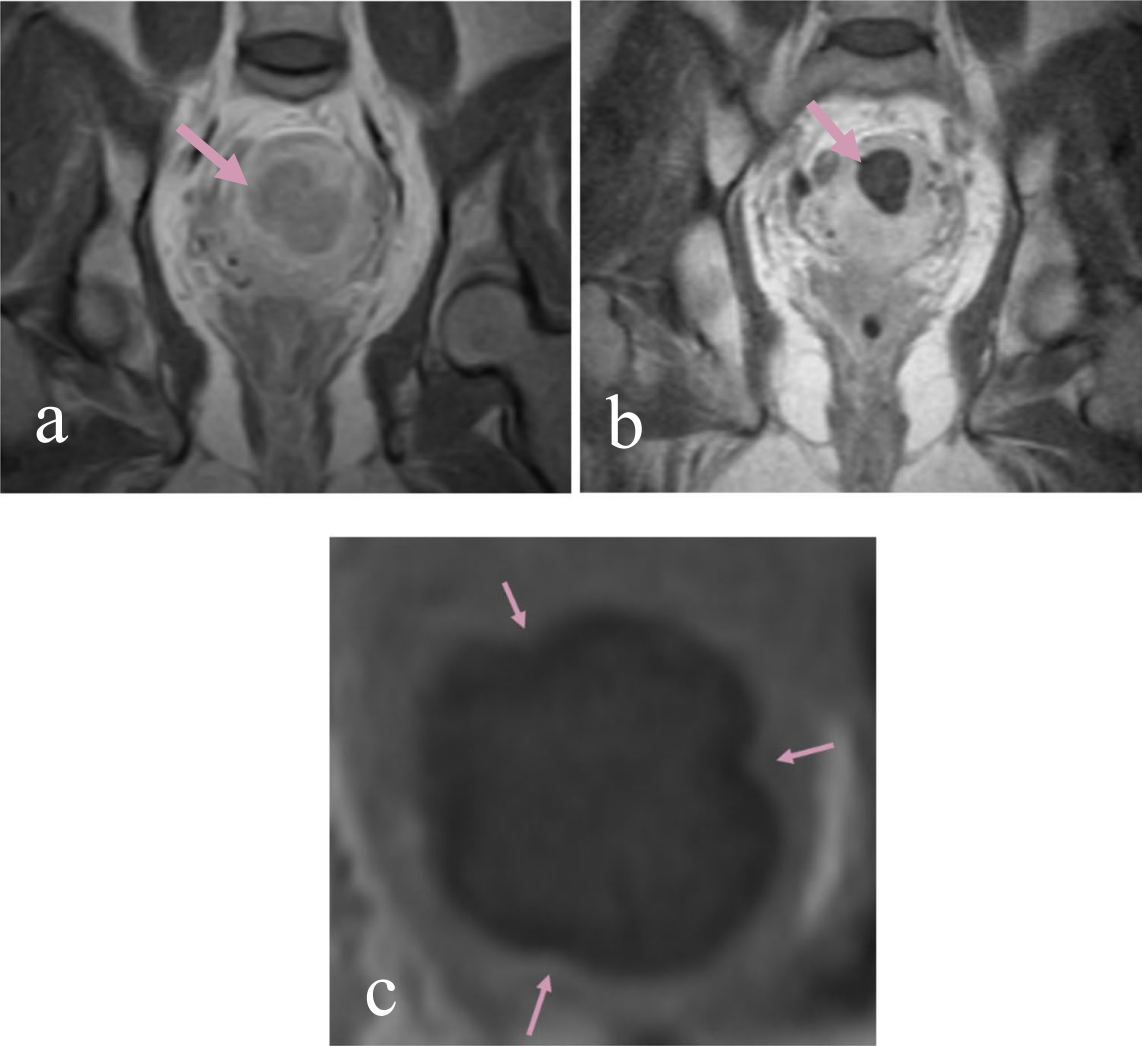
MRI—gadolinium-enhanced fat-suppressed T1-weighted images of a uterine fibroid **a** before the treatment—arrows point to the treated fibroid; **b** 12 months after the treatment; **c** 1 day after the treatment—arrows mark the sites at which sutures were placed with the PIFI system

A statistically significant reduction in volume of the treated fibroids was evident as early as 1 month after the procedure (Table 2). In the exploratory analysis, the effect endured over time and the effect size increased from a 16.0% volume reduction at 1 month to 60.4% at the end of the follow-up (Fig. 3).

**Table 2:** Post-procedural reduction in fibroid volume

Timing of assessment, (number of treated UFs)	Mean volume (cm^3^)	*SD*	95% CI	*P* value*	Mean % of volume reduction
Baseline (*N*^¶^ = 22, *n*^$^ = 16)	25.4	29.5	13.8, 39.2		
1-month FU (*N*^¶^ = 12, *n*^$^ = 9)	19.5	26.6	6, 40.4	0.030	28.9
3-month FU (*N*^¶^ = 19, *n*^$^ = 15)	15.8	22.2	6.2, 28.2	0.002	36.3
12-month FU (*N*^¶^ = 15, *n*^$^ = 11)	12.9	25.5	0, 25.8	0.008	60.4

*Student’s paired *t*-test, with a two-tailed distribution

^¶^Number of uterine fibroids

^$^Number of subjects, including the subject in whom a fibroid was observed on ultrasound and visualized during the procedure, but its volume could not be reliably estimated using MRI scans

In an ad hoc analysis, entries from the collected UFS-QOL questionnaires were used to calculate the questionnaire scores using the original scoring manual [35]. At the 3-month follow-up, the HRQOL total, Energy/Mood, Control, and Sexual Function domains scores were statistically significantly lower, i.e., better, than before the procedure (Table 3).

**Table 3:** UFS-QOL summary

Subscales	*N* respondents	Mean score	*SD*	*P* value compared to baseline*
Symptom severity				
Baseline	16	48.44	18.04	
1 M	11	44.60	18.49	0.729
3 M	15	42.92	21.57	0.258
12 M	11	37.50	25.58	0.098
HRQL** Total				
Baseline	16	55.17	23.27	
1 M	11	59.80	24.86	0.866
3 M	15	64.89	21.85	0.027
12 M	11	65.52	30.29	0.204
Concern				
Baseline	16	45.94	27.88	
1 M	11	43.18	35.59	0.132
3 M	15	58.67	28.81	0.066
12 M	11	52.73	33.94	0.416
Activities				
Baseline	16	60.04	23.26	
1 M	11	61.69	29.02	0.781
3 M	15	69.52	21.89	0.086
12 M	11	70.78	27.42	0.208
Energy/Mood				
Baseline	16	56.25	23.85	
1 M	11	64.29	24.80	0.648
3 M	15	65.00	24.31	0.043
12 M	11	69.81	31.03	0.176
Control				
Baseline	16	56.56	28.21	
1 M	11	57.73	29.70	0.622
3 M	15	67.33	22.35	0.021
12 M	11	69.55	36.64	0.191
Self-consciousness				
Baseline	16	59.90	29.85	
1 M	11	68.18	26.83	0.800
3 M	15	59.44	25.56	0.896
12 M	11	62.88	30.81	0.341
Sexual Function				
Baseline	16	46.88	38.32	
1 M	11	71.59	26.86	0.116
3 M	15	65.83	28.92	0.020
12 M	11	57.95	39.24	0.160

*Student’s paired *t*-test with a two-tailed distribution

**HRQL, health-related quality of life

Serious adverse events were not reported.

## Discussion

In this study, the PIFI system was effective in reducing fibroid volume, which showed a consistent and significant decrease up to the final study visit 12 months post-procedure.

Assessment of the UFS-QOL questionnaire responses collected from the study subjects at the 3-month follow-up, at which all but 1 subject completed the questionnaire, were also encouraging. Several health-related quality of life domains of the questionnaire showed a statistically significant improvement at this time. The effect was not durable (data not shown), but the overall impact of the PIFI system on the quality of life may well be underestimated, since not all eligible uterine fibroids were treated in each subject in this trial, to limit the exposure to the investigational treatment assessed in a feasibility study.

n 2014, the United States Food & Drug Administration (US FDA) issued an update to the safety communication on the risk of spreading unsuspected cancerous tissue, notably uterine sarcomas, beyond the uterus when using uncontained laparoscopic power morcellation [36–38]. The US FDA reaffirmed its views in 2020 [39, 40]. This development has undoubtedly caused profound changes in the standard practice of American gynecological surgeons and also affected their colleagues outside the United States [41–48] Although the odds of myomectomy compared with hysterectomy for leiomyoma remained unchanged, -a significant decrease in minimally invasive myomectomies was reported [49–52]. In an online survey, nearly half of the members of the American Association of Gynecologic Laparoscopists (AAGL) and of the American College of Obstetricians and Gynecologists Collaborative Ambulatory Research Network (ACOG CARN) reported an increase in their rate of laparotomy in view of the FDA warning [44]. Black women who were already more likely to have surgical interventions for fibroids [53] showed a significantly greater change in the odds of abdominal over laparoscopic myomectomy in comparison with the white population [54–60] This shift is concerning, as abdominal myomectomy is associated with significant morbidity, including excessive peri-operative blood loss, adhesion formation, pain, and/or impaired fertility [61, 62].

The minimally invasive options in existence include UAE, but its cost and availability limit its use [63], and extrauterine blood supply to a tumor may undermine the approach in certain cases [64]. The less established alternatives include MRI-guided focused ultrasound (MRgFUS) and radiofrequency fibroid ablation, which can be performed laparoscopically, transcervically, and transvaginally [52]. But these technologies either lack compelling evidence to support wide use or are limited in availability [52]. Development of other minimally invasive alternatives is clearly warranted.

Reduction in fibroid volume is infrequently reported in studies of myomectomy during which fibroids are removed [1] and effectiveness of the PIFI system presented in this work would be best appreciated in comparison with that reported for UAE, as the latter also brings about infarction of the tumor. This task may be challenging, in view of the differences between imaging modalities and duration of follow-up in the clinical trials involving UAE [1]. The low number of observations also could have contributed to over- or under-estimation of the effect size in some of these studies. Nonetheless, the dynamics of fibroid volume response in the randomized controlled EMMY trial [65] was similar to the incremental volume reduction observed in this work. In the EMMY trial, the fibroid volume reduction from baseline equaled 14.8%, 42.1%, and 54.5% at 1.5, 6, and 12 months after UAE [65]. In our study, sufficient data could not be collected at 6 months after the procedure, due to the travel restrictions during the pandemic. However, the effect of the PIFI system was nominally more pronounced early in the study, i.e., 28.9% at 1 month post-procedure, and numerically greater at the 12-month mark. Generalizability of the reported success in causing gradual shrinkage of the treated tumor is strongly supported by the fact that the study was conducted in the Dominican Republic. The majority of Dominicans have sub-Saharan African ancestry [66]. “Dominican” or “Dominica Islander” roll up to the “Black or African American” race category [67] of the United States Office of Management and Budget (OMB) *Standards for the Classification of Federal Data on Race and Ethnicity* [68] used for collection of ethnicity data in clinical trials [69]; and so does “Haitian,” although abundant genetic and self-identification differences exist between the inhabitants of Hispaniola [70–73].

Although PIFI builds on the same premise as UAE, there are several important differences between the modes of action of the two. Most importantly, UAE utilizes intra-vascular particles, blocks flow indiscriminately to all tissue distal to embolization site, i.e., the uterine arteries, thereby inflicting organ-wide ischemia and posing the risk of non-target embolization and, in rare cases, of pulmonary embolism. PIFI is intended to allow more precision by applying pressure to the fibroid and sparing the normal tissue. The PIFI system used in this trial was designed to apply pressure circumferentially on the fibroid surface, similarly to what occurs during labor. Uterus contraction during natural labor generates a pressure of 30–40 mmHg [74, 75] which exceeds the capillary blood pressure, halts the flow in uterine arteries, and leads to transient uterine ischemia [74, 76–80]. With the capillary blood pressure in the fibroid ranging 10.5–22.5 mmHg [75], exerting pressure of approximately 30–40 mmHg on the fibroid would occlude its blood flow, much akin to uterine contraction during labor. Unlike uterine contraction during labor, pressure exerted by suture(s) placed during PIFI procedure is envisioned to last days to weeks and lead to fibroid ischemia, infarction, and, eventually, necrosis followed by absorption. Another notable difference between PIFI and UAE is that by applying pressure to the treated fibroid, the PIFI system treatment outcome is not affected by variations in the fibroid vascularization known to predict UAE failure [64]. On the safety side, use of the PIFI system does not require ionizing radiation that is administered during UAE and involves exposure of the patient’s uterus and ovaries to a direct X‐ray beam [81]. This is particularly important to patients of childbearing age, as is the inapplicability of another UAE complication, premature menopause induced by non-target embolization of the ovarian parenchyma [82], to the PIFI system evaluated in this study. This was a feasibility study and it was subject to a number of limitations, both those inherent to the single-arm study design [83] and those levied by the COVID-19 pandemic which impeded efforts to adhere to the study visits schedule. While more rigorous investigation is needed, the results of this trial suggest that the design and mode of operation of the PIFI system make it an effective tool which may, with time and conditioned on availability of sufficient evidence, provide another minimally invasive alternative to the treatment of fibroids. In conclusion, the results of this clinical pilot of the PIFI system in pre-menopausal women suggest the device can be effective for its intended purpose. Larger trials will help confirm this premise and better establish the device’s safety and efficacy profile.

## Supplementary Information

Below is the link to the electronic supplementary material.

Supplementary file1 (MP4 228487 KB)
